# Shaft Fractures in Patients Requiring Primary or Revision Total Knee Arthroplasty Can Be Successfully Treated with Long-Stemmed Implants without Additional Fixation

**DOI:** 10.3390/jcm10214926

**Published:** 2021-10-25

**Authors:** Dariusz Grzelecki, Dariusz Marczak, Kamil Kwolek, Piotr Dudek, Marcin Tyrakowski, Łukasz Olewnik, Maria Czubak-Wrzosek, Jacek Kowalczewski

**Affiliations:** 1 Department of Orthopedics and Rheumoorthopedics, Centre of Postgraduate Medical Education, Professor Adam Gruca Orthopedic and Trauma Teaching Hospital, Konarskiego 13, 05-400 Otwock, Poland; darmarczak@poczta.onet.pl (D.M.); piotrek.dudek@gmail.com (P.D.); jackow@o2.pl (J.K.); 2 Department of Spine Disorders and Orthopedics, Centre of Postgraduate Medical Education, Professor Adam Gruca Orthopedic and Trauma Teaching Hospital, Konarskiego 13, 05-400 Otwock, Poland; kwolekamil@gmail.com (K.K.); marcintyrak@gmail.com (M.T.); czubakwrzosek@gmail.com (M.C.-W.); 3 Department of Anatomical Dissection and Donation, Medical University of Lodz, Żeligowskiego 7/9, 90-752 Lodz, Poland; lukasz.olewnik@umed.lodz.pl

**Keywords:** periprosthetic fracture, osteoarthritis, revision total knee arthroplasty, femur shaft fracture, tibial shaft fracture, stem extension

## Abstract

The aim of this study was to evaluate the bone union, complication rate, clinical and functional outcomes of long-stemmed total knee arthroplasty (TKA) in patients with periprosthetic femoral or tibial shaft fractures and in patients with femoral or tibial shaft fractures with coexisting advanced knee osteoarthritis (OA). This retrospective study comprised 25 patients who underwent surgery due to tibial or femoral shaft fractures: (1) with coexisting severe knee OA or (2) with a periprosthetic fracture requiring implant exchange. In all cases, fracture stabilization was performed intramedullary with the use of long-stemmed implants without the use of additional fixation material (plates, screws, or cerclage). Bone union was achieved in 22/25 patients (88%). One patient required revision with additional plate stabilization due to non-union, and asymptomatic partial bone union was observed in two cases. The group with periprosthetic fractures demonstrated good clinical (mean 73.1 ± 13.3) and moderate functional (mean 59.2 ± 18.8) outcomes in the Knee Society Scoring system (KSS). In the group with shaft fracture and coexisting OA significantly higher clinical (excellent results, mean 84.1 ± 11; *p* = 0.03) and functional (good results, mean 76.2 ± 20.6; *p* = 0.04) results were noted. There were no statistically significant differences in terms of range of motion (ROM) or complication rate between these two groups. One-stage TKA with a long-stemmed implant without the use of additional fixation material is an effective method for the treatment of femoral or tibial shaft fractures in patients who require joint replacement. Despite being technically demanding, the approach yields bone union and moderate to excellent clinical and functional outcomes with a relatively low complication rate.

## 1. Introduction

The gold standard for treating advanced knee osteoarthritis (OA) is total knee arthroplasty (TKA). However, the incidence of TKAs is increasing worldwide, accompanied by a rise in periprosthetic fractures. The estimated frequency of periprosthetic fractures after primary procedures varies from 0.3 to 2.5% [1,2], and it is related to the age and sex of the patient, fracture localization, time of occurrence, patient demographics and concomitant diseases [3,4,5]. In addition, the incidence is higher in revision knee arthroplasties (rTKA) than in primary procedures and may reach up to 8% [5,6].

The choice of treatment strategies depends on several factors, including fracture morphology, localization, bone quality, type of the used primary implant and its fixation to the bone [4]. Thus, every case should be particularly analyzed and the management matched individually. Several methods of treatment, including open reduction and internal fixation (ORIF) with plate and/or nail, and rTKA can be applied [7,8,9]. In some rare cases, in patients with loosened prosthesis, the fracture can occur at the level of the femur or the tibial shaft; it can also be found at the top of the prosthesis stem, where it is difficult and technically demanding to achieve stable fracture fixation [10]. 

In addition, some patients with severe knee OA also have concomitant extra-articular fractures, especially at the level of the femoral or tibial shaft. In such cases, the proposed treatment algorithms include two-stage procedures with internal fixation and subsequent TKA after bone union, or more rarely, one-stage osteosynthesis with TKA. However, two-stage procedures are characterized by a significantly longer treatment time, and this method is usually not acceptable for patients [11]. A new solution which has offered promise in some cases combines TKA with fracture stabilization using a long-stemmed prosthesis without additional hardware application [12,13,14]. A similar approach has been used with good results in patients with lower limb deformities and coexisting knee OA. In those cases, periarticular corrective osteotomies were performed and stabilized with long-stemmed TKA, with or without additional fixation [11,15].

The aim of this study was to evaluate the bone union and complication rate, as well as the clinical and functional outcomes of long-stemmed total knee arthroplasty (TKA) in patients with periprosthetic femoral or tibial shaft fractures and in patients with any femoral or tibial shaft fracture with coexisting advanced knee osteoarthritis (OA).

## 2. Materials and Methods

This retrospective study has been approved by the Institutional Review Board without the need to obtain informed consent from participants and was conducted in accordance with the ethical standards laid down in the 1964 Declaration of Helsinki.

The patients included in the analysis were operated on from December 2008 to September 2020 in a single orthopedic center. The inclusion criteria comprised femoral or tibial shaft fractures according to Müller et al. [16], in patients who required primary TKA (due to coexisting severe knee OA) or rTKA (due to implant loosening or malposition). The exclusion criteria were patients treated with additional fixation material such as plates, screws or intramedullary nails, two-stage revisions, resection arthroplasty, periprosthetic joint infections or infected pseudarthrosis or those with metaphyseal fractures.

**Figure 1:**
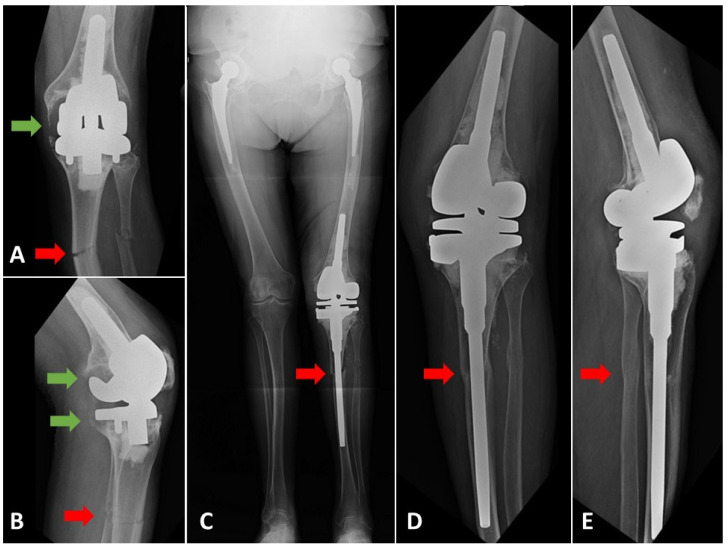
(**A**) AP and (**B**) lateral radiographs of a patient with the fracture of 1/3 proximal tibia and fibula shafts and loosened components of the knee prosthesis. (**C**) Postoperative, long-standing AP radiograph. Rotating-hinge revision knee prosthesis was used (MRH, Stryker, Mahwah, NJ, USA). (**D**) AP and (**E**) lateral radiographs of the same patient performed on the control visit 96 months after rTKA. Bone union was achieved. Green arrows indicate peri-implant bone osteolysis and loosening. Red arrows show the fracture level of the tibial shaft and bone union.

**Figure 2:**
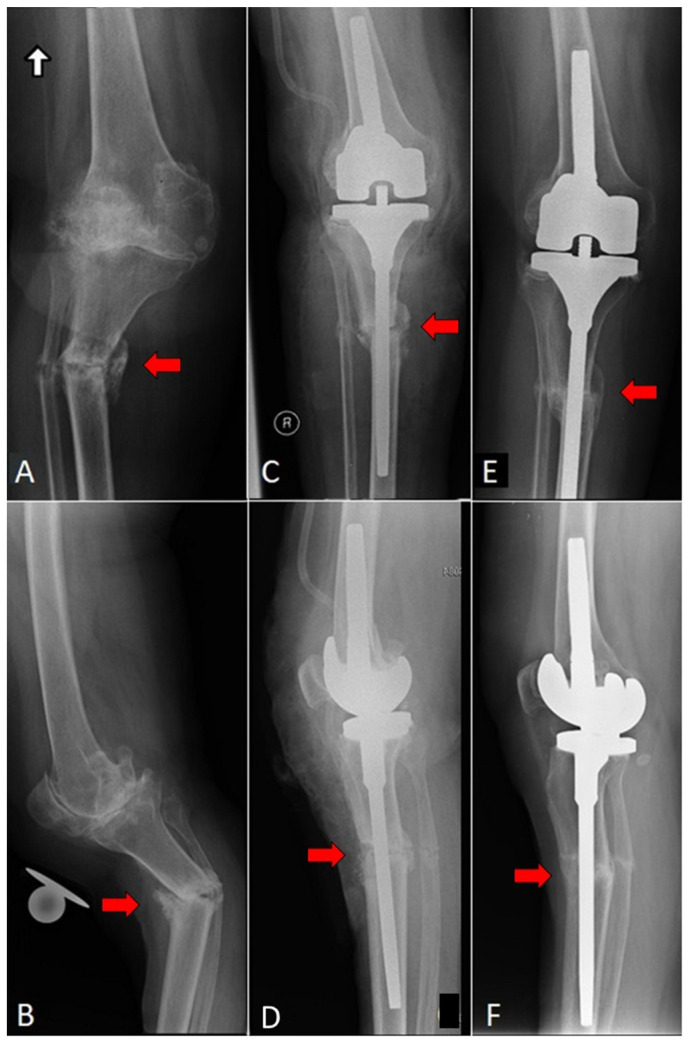
(**A**) AP and (**B**) lateral radiograph of the patient with 1/3 proximal tibial and fibular shaft fractures (pseudarthrosis) and coexisting severe knee osteoarthritis. Early postoperative radiographs (**C**) AP and (**D**) lateral (Scorpio TS, Stryker, Mahwah, NJ, USA). CCK knee implant with long tibial stem was used. (**E**) AP and (**F**) lateral radiographs of the knee performed.

Finally, 25 patients met the inclusion criteria and were selected retrospectively from the electronic database. These were divided into two groups depending on the preoperative diagnosis. The first group, known as the *periprosthetic fracture* group, included 12 patients with type III periprosthetic femoral shaft fractures according to Lewis-Rorabeck [17], or with type II or III tibial shaft fractures in accordance with Felix et al. [18], with a loosened or malpositioned implant (Figure 1). The second group, known as the *fracture with OA* group, comprised 13 patients with isolated shaft fractures of the femur or tibia with coexisting severe knee OA (Figure 2). In all cases, the bone union was assessed in outpatient visits, both clinically and radiologically, with the latter performed using plain radiographs in anteroposterior (AP) and lateral views [19]. Knee Society Score (KSS) was used for clinical and functional assessment [20]. 

All patients were operated on by three experienced knee surgeons from the same department. In every case, digital preoperative planning was performed with the use of OrthoView^TM^ (Jacksonville, FL, USA). Surgery was performed either with a medial approach in normal or varus alignment knees, or a lateral approach in valgus knees. In eight cases (four cases in each group) metalwork was removed during the same procedure. All of the patients received cemented TKA and detailed implant selection: constrained condylar knee (CCK) or constrained rotating-hinge (RH) was performed based on posterior capsule and collateral ligaments insufficiency (Table 1). The need for additional augmentation and/or metaphyseal sleeves or cones was decided during the surgery. Full weight-bearing was allowed on the first day after surgery, and was continued with the assurance of two crutches for six weeks, i.e., until the first outpatient visit.

Statistical analysis was performed with the use of Statistica 13.1 (Tibco Software Inc., Palo Alto, CA, USA). Normal distribution was checked with the use of the Shapiro–Wilk test. As the data were found to have a normal distribution, clinical and demographical continuous variables were presented as means (±SD). Differences between groups were evaluated using Student’s T-test. Dichotomous results were analyzed with the use of the χ2 (2 × 2, 3 × 2 and 4 × 2) test. Differences of *p* < 0.05 were regarded as statistically significant.

## 3. Results

**Figure 3:**
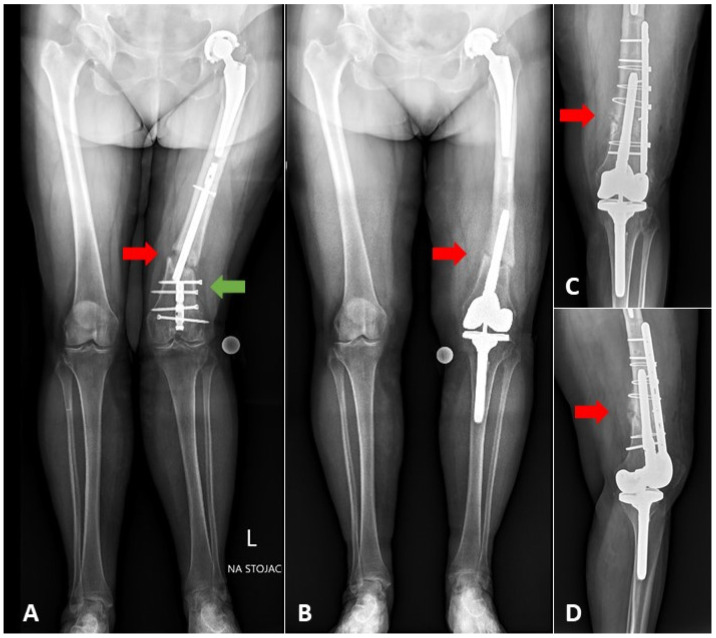
(**A**) AP long-standing radiograph of the patient with the fracture of 1/3 distal part of the femoral shaft, with the broken retrograde femoral nail (green arrow), and knee osteoarthritis; (**B**) long-standing radiograph performed nine months after the one-stage TKA with long-stem extension. Bone union was not achieved due to insufficient stability of fracture causing excessively short (64 mm from the tip of stem to fracture level) and undersized stem (13 mm stem diameter filling 68% of medullary canal in the narrowest point). Shorter femoral stem was used due to the presence of hip prosthesis ipsilaterally. (**C**) AP and (**D**) lateral radiographs after re-stabilization with the additional plate fixation. Red arrows indicate fracture level.

The mean age was 74.2 ± 7.4 years in the *periprosthetic fracture* group and 69.9 ± 8.9 years in the *fracture with OA* group (*p* = 0.12). The mean follow-up for both groups was 33.5 months (ranging from 9 to 114 months). Complete bone union was observed in 22/25 patients (88%). In one patient with a femoral shaft fracture and broken retrograde nail, a non-union was observed post TKA (Figure 3). This patient required another surgical procedure with additional plate stabilization. Partial bone union was confirmed in two other patients. 

The first one presented a Felix II periprosthetic fracture with contact to the top of long-stemmed prosthesis at the level of 1/2 middle of tibial shaft, while the second demonstrated posttraumatic knee OA, fracture of the 1/3 proximal tibial shaft, and poor bone stock. These patients did not report any pain in the fracture site after surgery, during the follow-up visit (35 and 12 months, respectively). Seven femoral and five tibial shaft fractures were observed in the periprosthetic fracture group, and four femoral and nine tibial shaft fractures in the fracture with OA group. In both groups, CCK implants and constrained RH prostheses were applied with a distribution of 7/5 in the periprosthetic fracture and 10/3 in the fracture with OA groups, respectively. No statistically significant differences were observed regarding the other demographical data. Similarly, no differences in the range of motion (ROM) and complication rate between periprosthetic fracture and fracture with OA groups were found (Table 2).

**Figure 4:**
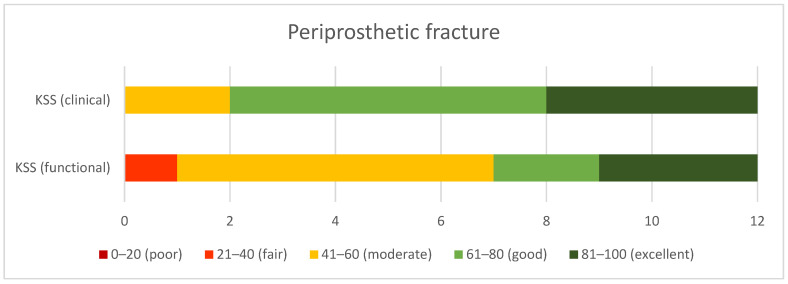
Clinical and functional outcomes in KSS in the periprosthetic fracture group.

**Figure 5:**
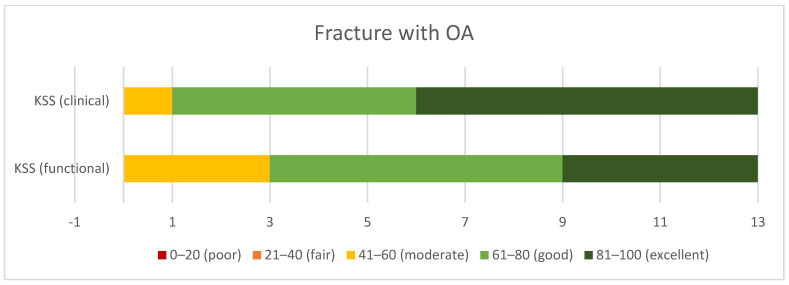
Clinical and functional outcomes in KSS in the group of patients with fracture and coexisting osteoarthritis.

According to the KSS system, the *periprosthetic fracture* group demonstrated good clinical (mean 73.1 ± 13.3) and moderate functional (mean 59.2 ± 18.8) outcomes, (Figure 4), while the *fracture with OA* group demonstrated excellent clinical (mean 84.1 ± 11), and good functional results (mean 76.2 ± 20.6) (Figure 5). The *fracture with OA* group demonstrated significantly higher clinical and functional KSS scores than the *periprosthetic fracture* group (*p* = 0.03 and *p* = 0.04, respectively).

## 4. Discussion

Although, periprosthetic fractures are relatively rare complications of TKA, surgical treatment is complex and technically demanding. Similarly, the management of fractures around knee joints in patients with coexisting OA and in those with pre-existing deformities is difficult and requires high surgical skills and experience [6,21,22]. In every case, precise preoperative planning is essential to achieve bone union, proper limb alignment and satisfactory clinical and functional outcomes [23]. Various methods of osteosynthesis have been proposed in surgical treatment strategies, such as single or double plating systems, intramedullary nailing (IM) and rTKA. However, recent reports emphasize that the final outcomes strongly depend on the fracture pattern and localization [6,8,24]. A study of periprosthetic tibial fractures found that Felix I fractures are associated with a higher risk of postoperative non-surgical complications, and that those treated with a proximal tibia replacement may cause the development of periprosthetic joint infections [24]. Bauer et al. emphasize that while functional results are good, periprosthetic tibial fractures demonstrated high complication and revision rates [25]. Their analysis, based on the French Society of Orthopedic Surgery and Traumatology (SoFCOT) classification [26], concluded that type B (fracture contact with keel or stem) has an especially poor prognosis. Periprosthetic femur fractures have been associated with substantial morbidity and mortality, regardless of fixation technique [27]. However, no consensus exists on whether plating or IM is a superior method of internal fixation. IM has been found to be associated with limb malalignment in the sagittal plane but with no differences in complication or reoperation rate [28]. Similarly, Gondalia et al. did not report a higher complication rate for either of these fixation methods; however, IM was associated with a slightly higher chance of non-union after plating and refracture [29]. In the case of implant loosening or malposition, rTKA is required independently for fracture fixation. Relative indications for rTKA are stated as poor bone quality or fracture comminution. A retrospective study found the ORIF group to have identified superior functional KSS results than the rTKA group in also higher revision incidence [30]. A difficult complication is the occurrence of fractures around the TKA with stem extension. Only a few studies have analyzed this with regard to fracture level, bone union and implant stability. We agree with Shin et al. that metaphyseal localization is often related to comminution and collateral ligament insufficiency, and the use of implants with higher constraints might be needed [14]. When the fracture site covers the stem extension level or bone shaft, it requires an individual approach and the use of rTKA with a longer stem, either with or without additional fixation. Subsequently, in ORIF, hardware failure requires another strategy and sometimes conversion to rTKA with a long stem [31]. In these rare cases, bone union is more difficult to achieve due to pseudarthrosis and soft-tissue damage.

TKA also represents a possible strategy for complex articular fractures, and allows weight-bearing with joint mobilization immediately after the surgery [13]. Unfortunately, most of the studies describing this method are case reports and case series, with short follow-ups, and most concern the elderly population [21,32,33,34,35]. Taking into account patient age, bone quality, and multiple comorbidities, TKA should be considered instead of ORIF due to the possibility for early mobilization and the lower chance of general complications [21,32]. In addition, single studies have found TKA to have encouraging results and better outcomes regarding secondary to ORIF failure [36]. TKA has also been associated with lower revision and complication rates, supporting it in the first-line treatment of fractures around the arthritic knee joint. Our clinical and functional scales results are in line with those of Wui et al., who received excellent short-term clinical outcomes in 8 of 10 patients after primary TKA for fractures around the knee joint without increasing the risk of infection [37]. 

**Figure 6:**
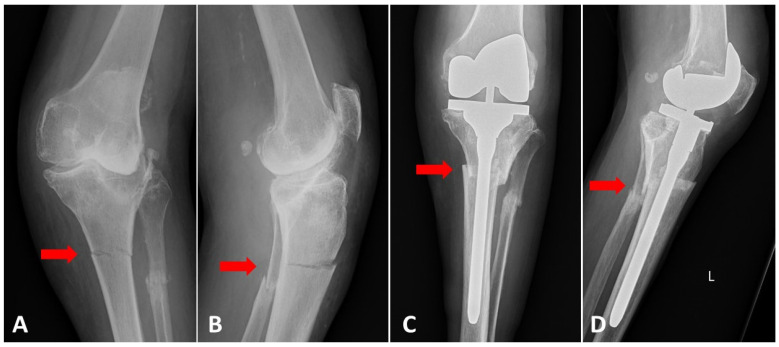
(**A**) AP and (**B**) lateral radiograph of the patient with the fracture of 1/3 proximal of the tibial shaft with coexisting severe osteoarthritis and valgus knee. Postoperative (**C**) AP and (**D**) lateral knee radiographs. The fracture was fixated intramedullary with the use of a well-fitted long-stemmed TKA. Postoperative radiograph indicates that the use of 4 mm tibial offset on the lateral site direction could improve the alignment of bone fragments.

Our study raises the rare problem of the femoral or tibial shaft fractures (acute or pseudarthrosis) in patients with periprosthetic fractures and those with coexisting advanced knee OA. Present findings indicate that one-stage TKA with osteosynthesis on a well-matched, uncemented prosthesis stem allows sufficient stabilization of bone fragments for bone union and weight-bearing directly after surgery. However, this method requires high-grade surgical skills and experience combined with meticulous preoperative planning. In some cases, the use of metaphyseal cones, sleeves, augmentation and stem offsets are necessary to achieve implant stability, joint line restoration and axial alignment of the limb (Figure 6). 

Our study has some limitations which should be considered before implementing the method in clinical practice. The first and the most evident is the small group of patients; however, the number is not only comparable to those used in previous studies, but is larger than most. Nevertheless, the group size was sufficient for simple parametric analysis and allowed certain trends, especially KSS and complication rates, to be confirmed. In addition, it is difficult to compare separated groups from a functional point of view due to differences in preoperative diagnosis, bone quality and deficits, fracture site and performed procedures (primary or revision TKA), and results cannot be compared between fracture levels on the bone shaft. A second limitation is the heterogeneity in terms of fracture type: altogether eight patients were admitted to the hospital with acute fracture and sixteen had pseudarthrosis that occurred mostly as a result of the instability and failure of prior osteosynthesis hardware. This factor may strongly influence the postoperative bone union, ROM, clinical and functional outcomes. Lastly, different types of prosthesis were used (CCK or RH). The need to use a prosthesis with increased constraints to account for posterior capsule and collateral ligaments insufficiency influences the final results and patient satisfaction.

## 5. Conclusions

One-stage long-stemmed TKA, without the use of additional osteosynthesis material, appears to be a promising option for the treatment of femoral or tibial shaft fractures in patients who require joint replacement. The use of uncemented stems allows sufficient fracture stabilization and bone union in 88% of cases. Additionally, moderate to excellent clinical and functional KSS results were obtained, which support the use of the method in the discussed groups with significantly better outcomes observed in the shaft fractures in patients with coexisting OA. However, we are aware that this method requires further investigation and more extensive analysis should be performed on a larger group of patients before application in routine clinical practice. 

## Figures and Tables

**Table 1:** Type of implant used in the study groups.

	Periprosthetic Fractures (*n* = 12)	Fractures with Knee OA (*n* = 13)
**Constrained condylar knee** **(CCK)**		
Triathlon TS (Stryker, Mahwah, NJ, USA)	5	7
PFC Sigma TC3 (DePuy Synthes, Raynham, MA, USA)	1	2
Vanguard 360 (Zimmer Biomet, Warsaw, IN, USA)	1	-
Scorpio TS (Stryker, Mahwah, NJ, USA)	-	1
**Constrained rotating-hinge** **(RH)**		
MRH (Stryker, Mahwah, NJ, USA)	5	3

The types of implants (level of constraint) used in the study are marked in bold.

**Table 2:** Demographic and clinical data in the analyzed sub-groups of patients. Continuous variables are presented as means (±SD).

	Periprosthetic Fractures	Fractures with Knee OA	*p*-Value
No.	12	13	-
Gender (M/F)	1/11	1/12	0.95 *
Age (years)	74.2 (±7.4)	69.9 (±8.9)	0.12 **
Prosthesis type			0.32 *
*semi-constrained (CCK)*	7	10	
*constrained (RH)*	5	3	
Fracture level (shaft)			0.07 *
*middle 1/3 femur*	2	0	
*distal 1/3 femur*	5	4	
*proximal 1/3 tibia*	3	9	
*middle 1/3 tibia*	2	0	
Type of fracture			0.06 *
*acute fracture*	6	2	
*pseudarthrosis*	6	11	
Bone union rate			0.61 *
*total*	11	11	
*partial*	1	1	
*non-union*	0	1	
Hardware in situ before TKA			0.89 *
*Yes*	4	4	
*No*	8	9	
Concomitant diseases			
*Rheumatoid arthritis*	2	1	0.59 *
*Diabetes*	1	2	0.59 *
Stem length (above or below the fracture level) (mm)			
*Femur*	109.3 (±31.1)	123.7 (±55.2)	0.6 **
*Tibia*	124.6 (±24.2)	125.5 (±22.9)	0.94 **
Stem diameter (% of filling the medullary canal in the narrowest point)			
*Femur*	95.5 (±4.4)	85 (±14.4)	0.1 **
*Tibia*	93.7 (±10.1)	90 (±8.4)	0.46 **
KSS (clinical)	73.1 (±13.3)	84.1 (±11)	0.03 **
KSS (functional)	59.2 (±18.8)	76.2 (±20.6)	0.04 **
ROM (mean, range)			
*Extension*	0°	0.77° (±2.8°) From 0° to −10°	0.35 **
*Flexion*	96.7° (±26°) From 25° to 120°	93.6° (±26.3°) From 30° to 120°	0.85 **
Complications			
*Surgical site infection*	2	1	0.59 *
*Arthrofibrosis*	1	1	0.95 *
*Delayed union*	1	2	0.59 *
*Non-union*	1	0	0.33 *

* χ^2^ test. ** Student’s *t*-test. Statistically significant differences were marked in bold.

## Data Availability

The datasets generated and analyzed in the current study are available from the corresponding author on reasonable request.
